# Performance evaluation of humpback whale-inspired shortboard surfing fins based on ocean wave fieldwork

**DOI:** 10.1371/journal.pone.0232035

**Published:** 2020-04-21

**Authors:** David E. Shormann, Marc in het Panhuis

**Affiliations:** 1 DIVE, LLC, Haleiwa, HI, United States of America; 2 Surf Engineering Association, Kiama Downs, NSW, Australia; 3 Surf Flex Lab, Australian Institute for Innovative Materials, University of Wollongong, Wollongong, NSW, Australia; Texas A&M University System, UNITED STATES

## Abstract

We present field results revealing improved surfing performance when a novel approach (“Real Whale”, RW) is used for applying several of the humpback whale’s passive flow control mechanisms, including tubercles, to surfboard fins. It is also the first study presenting evidence of dynamic performance of tubercled designs rotating on all three axes. We evaluated low aspect ratio, thruster-style 3-fin configurations used in high-performance surfing. Fieldwork involved surfing almost 2,000 ocean waves from around the world, comparing standard commercial fins with straight leading edges to RW fins. We collected surfing data from instrumentation attached to surfboards, including GPS and 9-axis motion sensors. Eighteen turn performance values were measured and calculated, including novel, surfing-specific rotational power coefficients. ANOVA revealed surfers using RW fins showed significant improvements in power generation compared to when they used standard commercial fins. Turn rates using RW fins also improved, although not significantly. We found using RW fins allowed a skilled surfer to improve their surfing performance relative to a professionally ranked surfer.

## Introduction

Fish and Battle’s 1995 paper [1] sparked great interest in humpback whale flipper passive flow control, and in particular their leading-edge tubercles, believed to be the key to explaining their incredible agility. Bushnell and Moore [2] were the first to suggest tubercles could play a role in flow control over the humpback flipper. Since the Fish and Battle paper [1], the standard biomimetic research method includes flow testing of highly idealized wing planforms focused almost exclusively on the number of leading-edge tubercles and/or their shape. An extensive review by Aftab et al. [3] revealed that, almost without exception, the tubercle shape chosen is a periodic, sinusoidal pattern, where the amplitude and wavelength are kept constant or varied only in proportion to chord length.

**Fig 1:**
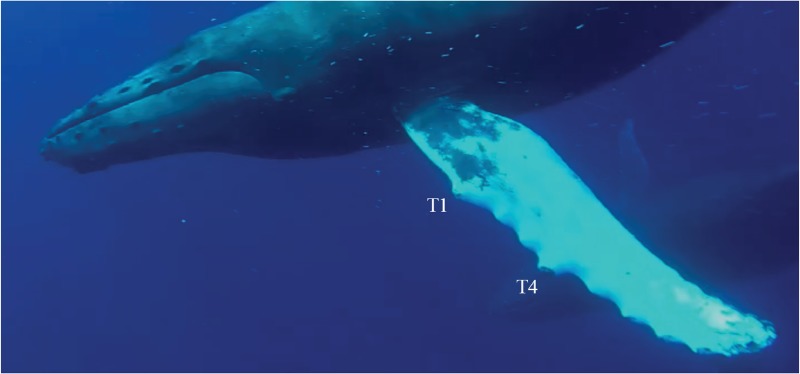
Photograph of a typical humpback whale pectoral fin, or flipper. T1 and T4 denote the first and fourth tubercles. Image credit: David E. Shormann.

On actual humpback flippers, however, leading edge tubercles follow a non-periodic pattern [4]. For example, as seen in Fig 1A, the first and fourth tubercles are noticeably larger than the rest. Furthermore, tubercles are not the only important features. Others include, but are not limited to, trailing edge crenulations or serrations, and a tapered spanwise profile. The tapered design of the humpback flipper is not unlike the design of a wind turbine rotor blade, and possibly for similar purposes related to thrust and torque optimization [5], as well as flow control [6]. Trailing edge serrations are known to provide benefits related to reduced noise and drag, and increased lift at high angles of attack [7].

The review by Aftab et al [3] expressed concern over the methods used to select tubercle geometry. Many studies mention the importance of tubercle geometry, but offer little explanation for their own selections [3, 8–9]. Others take their design cues from nature [10–17]. However, these studies mostly involve a bottom-up, tubercles-only approach, where the humpback’s average tubercle amplitude and wavelength is studied [10–11]. Some also include the natural flipper’s spanwise tapering pattern [12–15], or a NACA 63–021 airfoil cross section that mimics the natural humpback cross section [16–17]. These studies all reference measurements made by Fish and Battle [1] of an actual humpback flipper.

Shormann and Panhuis [6] were the first to use a top-down approach incorporating the non-periodic pattern of humpback tubercles and other features mentioned previously. Compared to designs with a smooth leading edge and another with tubercles, their top-down flipper design exhibited improved performance.

It is well-known that humpback whales (*Megaptera novangelaie*) use their pectoral fins in two primary ways: to generate lift and maneuver [18–20]. While they use their flippers as hydrofoils to ascend and descend, they also generate lift by rotating one or both flippers anteriorly, and to a lesser extent, short-duration flapping. They use rotation especially when they want to ascend or dive quickly. For example, during lunge feeding, they will often rapidly rotate their fins anteriorly in the plane of motion, generating massive amounts of lift [21–22]. Rapid rotation of their long (up to 6 m) flippers requires a design that maximizes lift while minimizing volume, thereby minimizing moment of inertia.

With rotational flipper tip speeds estimated near 20 m/s [4], it is apparent the humpback’s unique flipper geometry helps control flow under conditions with a high Reynolds (Re) number, and also a highly turbulent environment. Surface conditions in the open oceans where humpbacks roam is almost never laminar. Not only that, humpbacks often feed in tight formation [18–20], and spring mating rituals consist of fast-swimming groups of several males closely pursuing a single female [23].

A humpback’s survival depends on controlling its massive body amidst fast moving, turbulent flow [21]. If their flippers do not perform maximally in a variety of conditions, they lose because they cannot capture prey. It is well-known that surfers will not win during surfing competitions if their equipment (e.g. fins) does not perform maximally. Surfboard and surfing fin hydrodynamics [24–27], and surfer performance [28–33] have been investigated. However, reports combining studies coupling fin design with surfer performance are scarce [6, 34].

While some have recognized the potential benefits of tubercle applications to surfboard fins, efforts are mostly limited to untested hypotheses and subjective experience. New Zealand surfboard shaper Roy Stuart designed a 3D-printed single fin that some surfers feel has reduced drag [35]. Although performance results are unknown, 11-time world surfing champion, Kelly Slater, was seen in recent years testing some tubercle applications [36]. Also, it should be noted that the notches on Slater’s fins were more like grooves than tubercles.

Shormann and Panhuis [6] made the first objective investigation of tubercled surfboard fins, comparing a top-down, longboard single fin prototype to a standard design with a straight leading edge. Computational fluid dynamics(CFD) revealed the prototype had improved performance, as mentioned previously, including reduced drag, and higher lift/drag. The CFD results were validated in the field, where longer rides and faster speeds were achieved when surfing the tubercled prototype in ocean waves on the North Shore of Oahu.

While [6] focused only on speed and distance of a longboard single fin, [37–39] speculate on the possible control benefits of tubercled designs for shortboard fins, such as during a cutback maneuver, when fins undergo rapid changes in angle of attack. Tubercled designs are known to provide benefits of delayed stall and gradual stall [13, 39]. These phenomena were observed in CFD results performed on the humpback whale-inspired longboard single fin [6].

**Fig 2:**
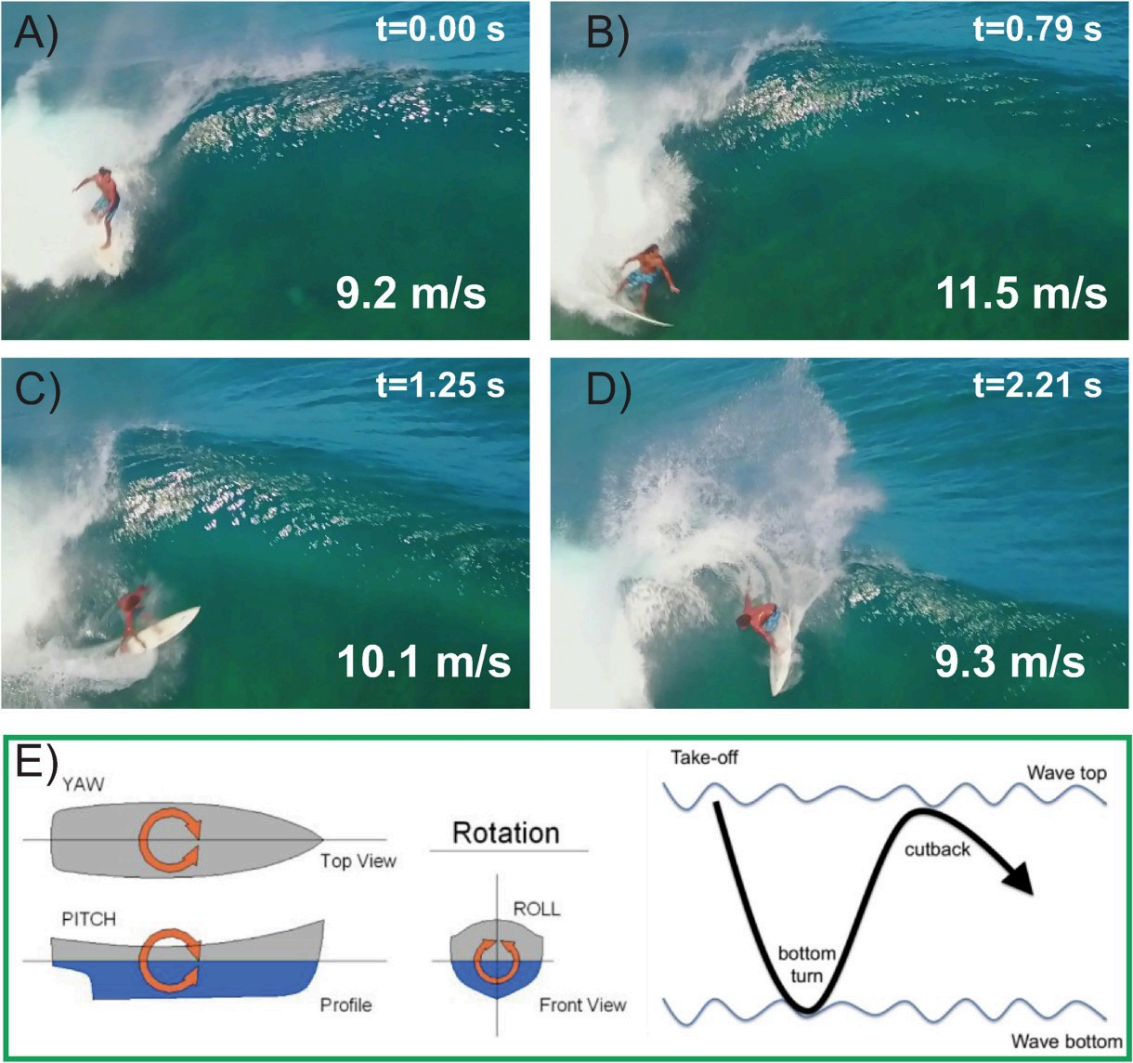
Bottom turn (BT) and cutback (CB) maneuvers. A) Surfer drops into a wave at t = 0, with speed 9.2 m/s. B) Bottom turn maneuver at t = 0.79 s with speed = 11.5 m/s. Note speed gain compared to A), and how surfer has rolled board onto the “back side.” C) Transition to cutback at 1.25 s, with speed 10.1 m/s. Speed drops (compared to C) as surfer pitches board and climbs wave face. D) Cutback maneuver, or top turn at t = 2.21 s. Note large amount of spray generated, and how board is now rolled onto the “front side.” The transition from back side to front side delineates the bottom turn from the cutback maneuver. See also Fig 4 and S1 Video for more details of the turn represented in A-D). E) Schematic representation of surfer’s trajectory depicted in A-D).

Like wings, surfboard fins provide a lift force as they are canted at an angle of attack during turning maneuvers. During a rapid and sharp cutback (See Fig 2), the angle of attack is high, and could lead to stall of the fins and loss of control in the turn. In surfing parlor, stall is referred to as “release.” If the tubercles cause this “release” to be slightly delayed and less abrupt, this could result in a smoother, more stable cutback (CB) maneuver and improved turn performance.

A cutback maneuver is complicated, requiring strength, agility, balance and timing, to name a few. To begin, surfers drop down on a wave, perform a bottom turn (BT) maneuver, and then transition to a powerful top turn, or cutback maneuver (Fig 2). In Fig 2, the surfer begins with his back facing the wave. In Fig 2A and 2B, the side of the board submerged in the water, and closest to the surfer’s posterior, is referred to as the “back side.” As the surfer transitions through the cutback to Fig 2D, the surfer rotates the board to its “front side.” The power generated during this cutback can be a significant factor in a surfing contest, as powerful turns with high rotation rates tend to impress the judges. Gately et al [34] concluded more skilled surfers typically make more powerful turns.

Shortboard yaw is the most visible rotation to judges and spectators, although pitch and roll are also important (See Fig 2). Surfing therefore provides a setting for analyzing dynamic performance of tubercled designs rotating on all three axes. To date, all studies on tubercled designs focus on only one or two axes of rotation.

In this paper, we detail an approach to quantify field performance of surfboard fins during high performance maneuvers like cutbacks. Our approach is based on analyzing data gathered by surfers of various skill levels testing our fins under real-life conditions, i.e. surfing ocean waves. In particular, we compare the surfing performance of 3-fin “thruster” configurations with tubercles (inspired by humpback whale passive flow control mechanisms) to standard commercial thruster configurations with straight leading edges.

## Materials and methods

### Fin designs

**Fig 3:**
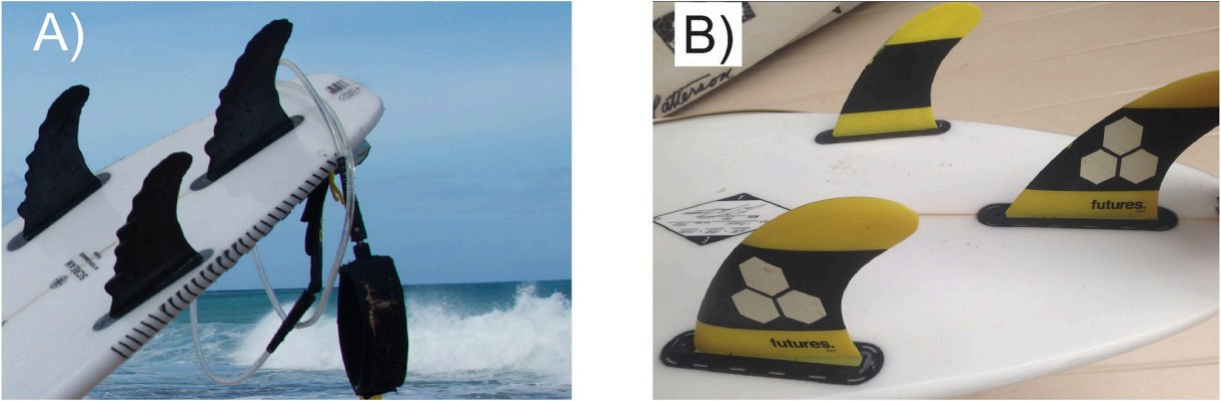
Control and RW fin configurations. Shortboards with A) humpback whale-inspired RW fins, and B) commercially manufactured control fins. Fin dimensions listed in Table 1. Image credit: David E Shormann.

The humpback whale-inspired, or real whale (RW) prototype fins (Fig 3A) were designed using the OnShape CAD program, and possessed one or more embodiments described in [4]. A composite-based Additive Manufacturing (CBAM) method by Impossible Objects (USA) was used to 3D print the prototype fins. Their carbon fiber/PEEK layering method allowed for prototypes of sufficient strength and durability comparable to industry standards. The fins were post-processed by sealing with 2-part XCR epoxy (Easy Composites LTD), sanded and polished.

Control fins (Fig 3B) were standard, “dolphin style” thrusters and center fins with straight leading edges manufactured by Futures Fins (USA). Table 1 lists make/model and dimensions of control and RW fins. Control and RW fins had similar sweep, as well as base length, height and area (Table 1). The treatment, or RW fins are basically the control (C) fins, modified with humpback flipper embodiments per [4].

**Table 1:** Surfer, surfboard, and surfing fin specifications. In shortboard performance surfing, surfers choose their fins and surfboards based on many factors, including experience, body size, and skill level. For example, Participant 3, a larger, more powerful surfer, would struggle to maintain buoyancy and control using Participant 1’s smaller board.

	Participant 1	Participant 2	Participant 3	Participant 4
Skill level ranking per [42]	6	7	8	9
Participant age(years)	17	21	32	-
Ranking description per [42]	Intermediate, skills of 5, plus able to execute occasional advanced maneuvers	Expert, able to execute advanced maneuvers and surf waves > 4.0 m	Expert, skills of 7, plus able to execute multiple advanced maneuvers on waves > 4.0 m	Top 44 surfers in world
Control fin make/model	Tokoro Honeycomb/Futures Fins LLC	NA	AM2 Honeycomb/Futures Fins LLC	-
Control center fin: base length/height/area (mm/mm/mm^2^)	115/116/9,445	NA	118/114/9,884	-
Control thruster fin: base length/height/area (mm/mm/mm^2^)	115/116/9,445	NA	118/120/10,300	-
RW Center fin: base length/height/area (mm/mm/mm^2^)	114/116/9340	114/116/10,452	114/116/10,452	-
RW 6° cant thruster fin: base length/height/area (mm/mm/mm^2^)	114/116/9479	114/116/9479	114/116/9479	-
Surfboard make/model	Eric Arakawa Amplifire	Schaper Pro Model	Eric Arakawa Scream	-
Surfboard length/width/thickness (cm), [volume in L]	178/47/6.0 [25.9]	185/48/6.3 [29.7]	188/50/6.7 or 198/48/7.0 [33.0]	-
Surfer + board moment of inertia, calculated per [34], (kg m^2^)	20.6	28.5	30.9–33.9	26
Surfer mass (kg)	73	93.2	98	~87.6

### Fin placement in surfboard

Surfboards may have no fins, or 5 or more fins. We chose the industry standard for performance shortboard surfing, a 3-fin configuration (Fig 3A and 3B) invented by Simon Anderson in 1980 [40]. 3-fin sets consisted of a symmetrical, 50/50 center fin, and two side, or “thruster” fins. Standard thruster fins are asymmetrical, described as 0/100, and flat or slightly concave on the side facing the center when inserted into a surfboard. The side thruster fins were canted by 6° cant, consistent with commercial standard practice. The center fin is mounted perpendicular to the board, at 0° cant. Thruster fins are also not parallel to the center fin, but are angled or “toed-in” between 2 and 3° so they point towards the board’s nose. Cant, toe-in and other thruster fin schematics are described in more detail in [41]. The 3-fin configuration is often preferred for surfing in more powerful wave conditions. In fact, Simon Anderson devised the configuration because of the lack of control his two-fin configuration provided in more powerful waves [40].

### Fin performance in ocean waves

Fin performance was evaluated while four participants of varying surfing expertise performed standard surfing maneuvers on ocean waves between 2015 and 2019. Participants ranged in skill level from intermediate to top ranked professional, per the ranking system developed by [42]. An overview of the participants and their surfing equipment is detailed in Table 1. Ethical clearance for field research was obtained from the University of Wollongong Human Research Ethics Committee under Ethics Number 2017/174. Written consent was obtained from each participant, with parental consent obtained for the one minor-aged participant. The data of participant 4 (WCT, a professional surfer on the World Surf League (WSL) Men’s Championship Tour) was made available by the manufacturers of the commercial tracking system (TraceUp, USA) for comparison to prior research on 3D-printed surfboard fins [34]. Dimensions of the surfboard, fins, and the identity of participant 4 were not disclosed to us.

A typical board and thruster fin setup is shown in Fig 3. Due to their larger size and strength, Participants 2 and 3 used a RW center fin with a slightly larger area than Participant 1 (see Table 1). Both the control and RW setup consisted of two thrusters and a 50/50 center fin. Control thrusters were flat on the inside, and RW’s were slightly concave. On occasion (less than 10%), an RW center fin was used by Participants 1 and 3, but the setup was considered a control since the majority of the fins (2 of 3) had smooth leading edges.

A commercial tracking system (TraceUp, USA), with 9 inertial sensors and a GPS (10 Hz sampling rate) was used by participants 1–4 to monitor and quantify the performance of the fins during each surfing trial. The tracker yielded angles (degrees; yaw, pitch, and roll, Fig 2), linear and rotational speed (m/s and rad/s; maximum and average), and power magnitude (a dimensionless number on a scale from 0 to 10 assigned by the tracking system) during characteristic surfing maneuvers (bottom turn, BT and cutback, CB Fig 2).

The tracking system returns GPS data (session location, time, distance and speed information), number of waves and turns (maneuvers), and board roll, pitch and yaw angles. Details on GPS accuracy are found in [43]. Table 2 provides a description of the various outputs produced by the tracking system. Tracking system measurements were used to calculate rotation rates and powers. Note, the tracking system is mounted on the bow of the surfboard, and collects roll, pitch and yaw data for the board (but not the fins). Hence, when the board rolls, the fins roll. When the board pitches, the fins yaw, effectively changing their sweep angle. When the board yaws, the fins pitch, effectively changing their angle of attack.

**Table 2:** Descriptions of measurements recorded from TraceUp^TM^ datalogger on cutbacks and bottom turns and downloaded by UOW’s algorithm. See also Fig 2 for a description of bottom turn and cutback maneuvers. The bottom turn occurs first, followed by the cutback.

**Bottom turn angle**	Yaw angle generated during bottom turn.
**Bottom turn offset**	Unix Epoch Time stamp for start of bottom turn.
**Bottom turn duration**	Time in seconds to generate bottom turn angle.
**Bottom turn roll angle**	Angle board rolls during bottom turn.
**Bottom turn speed gain**	Change in speed between start and end of bottom turn.
**Bottom turn initial speed**	Speed at start of bottom turn.
**Cutback angle**	Angle board yaws during cutback, or top turn.
**Cutback power**	Dimensionless number (scale 0–10) assigned by TraceUp^TM^ software rating how large or impressive the maneuver is.
**Cutback speed**	Mean speed during cutback.
**Cutback offset**	Unix Epoch Time stamp for start of cutback and end of bottom turn.
**Cutback duration**	Time in seconds to generate cutback angle.
**Cutback roll angle**	Angle board rolls during cutback.
**Cutback pitch angle**	Angle board pitches during cutback.

Participants 1–3 switched monthly between using prototype RW fins (Fig 3A) and high-quality commercial fins (Fig 3B). In addition to tracking system data, ocean conditions during each surf session were recorded (wave height, wave period, wind speed and direction). The surfing location, i.e. *surf break*, was also recorded. Various forecasting models were used to collect ocean data, which was cross-checked with participant observations. Ocean data collected on wave height (h) in meters and period (p) in seconds were used to predict wave power (WP) in kW • m^-1^ using [44]

$$\mathrm{WP}=0.5h^{2}p.$$ (1)

ANOVA was used to compare overall treatment (RW) versus control (C) measurements, with p-values < 0.05 considered significant. Participants 1–3 were also analyzed individually to discern whether fin type improved their performance relative to a top ranked professional (Participant 4). A large sample size (number of waves surfed) was collected from a variety of locations to minimize the dependence on skill level, wave power, and surf break [27, 42, 45–47].

## Results and discussion

### TraceUp^TM^ measured data analysis

**Fig 4:**
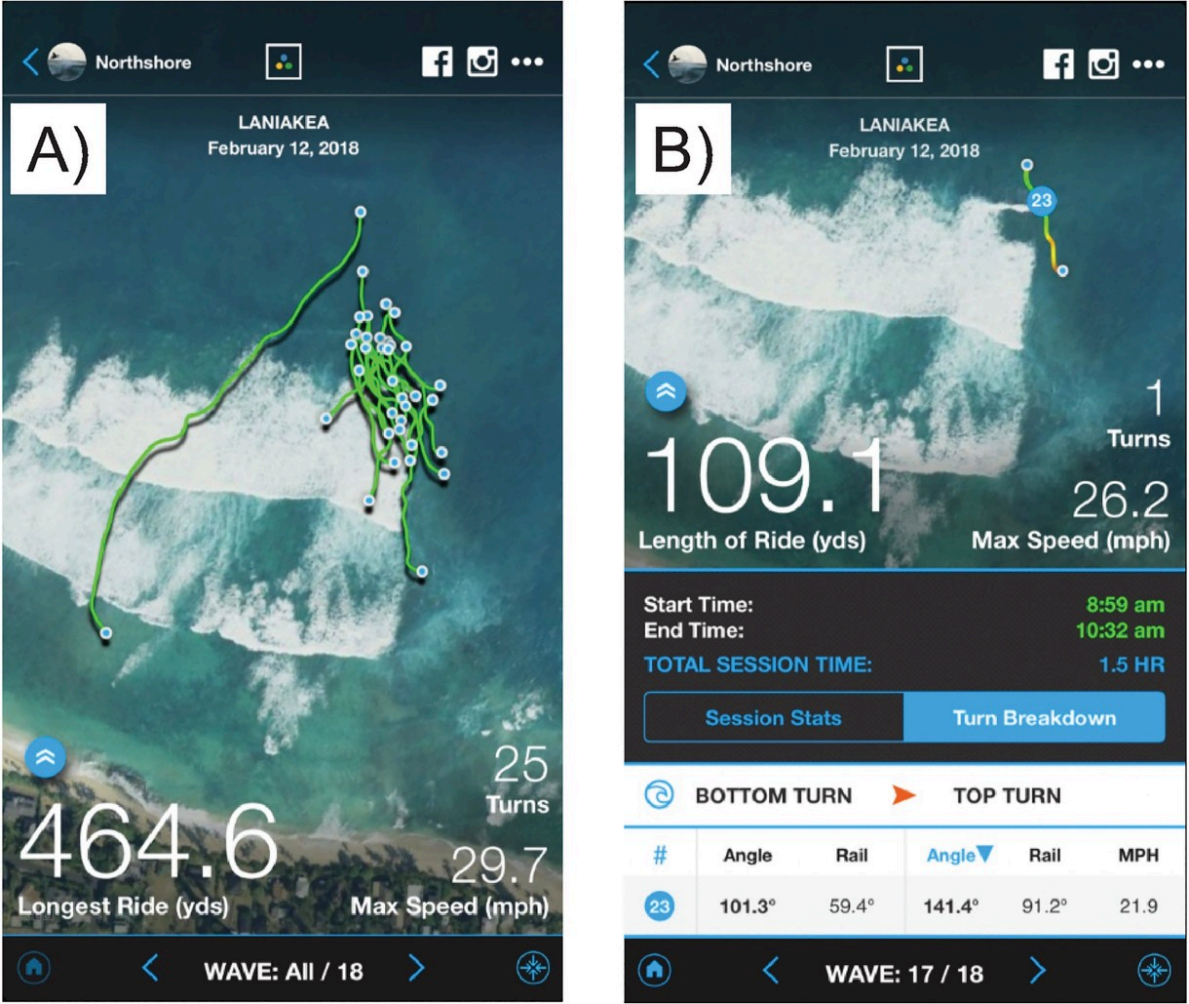
GPS tracks and TraceUp App details of a typical surfing session. A) GPS tracks and other details of 18 waves surfed in an actual field research surfing session. B) GPS track of wave 17, identifying details of Turn 23, which is also featured in Fig 2A–2D and S1 Video. Note the TraceUp App describes a *cutback* as a *top turn*.

Participants 1–4 performed a total of 2,060 cutback maneuvers on 1,920 waves surfed in 146 sessions as measured by the tracking system. Table 3 lists mean values of measured data used for the turn performance calculations listed in Table 4. See Table 2 for a description of measurements recorded by the datalogger. Fig 4 provides details of tracking system data collected during a typical surfing session. See also S1–S3 Videos for examples of turns recorded during field research.

**Table 3:** Summary of fieldwork data. Values shown are means ± 95% confidence intervals. Each surf session consists of one or more waves ridden. When a wave is ridden the surfer may complete one or more turns. Session data includes overall mean session speed, and overall mean wave power calculated per Eq 1. Waves data includes mean max speed and mean distance surfed on a wave. Turns data includes means of the remaining data, where CB and BT indicate cutback and bottom turn, respectively. All values, except mean waver power, are measured by the TraceUp^TM^ datalogger. Raw data is found in S1–S3 Files.

	Participant				Fins		
Fieldwork summary	1	2	3	4 (WCT)	Control	RW	p-value
**Skill level ranking per [25]**	6	7	8	9	-	-	-
# sessions	50	8	51	37	55	54	-
**Mean wave power (kW/m)**	10±3	25±10	17±3	-	16±3	13±3	0.184
**Mean session speed (m/s)**	4.6±0.1	6.1±0.5	6.0±0.3	5.7±0.3	5.5±0.3	5.2±0.3	0.148
# waves	696	98	578	548	693	679	-
**Max speed (m/s)**	7.3±0.1	10.1±0.3	9.2±0.2	9.7±0.2	8.5±0.2	8.1±0.1	**0.002**
**Distance (m)**	60±3	85±8	72±4	67±4	73±3	62±3	**< .001**
# turns	658	110	713	579	815	666	-
**CB yaw angle (rad)**	2.1±0.04	2.6±0.1	2.7±0.05	3.1±0.08	2.4±0.05	2.4±0.05	0.690
**CB roll angle (rad)**	0.8±0.02	1.1±0.08	1.1±0.03	1.3±0.04	1.0±0.02	1.0±0.03	.704
**CB pitch angle (rad)**	0.5±0.02	0.6±0.05	0.6±0.02	0.8±0.03	0.6±0.02	0.6±0.02	**0.008**
**BT yaw angle (rad)**	1.2±0.04	1.6±0.1	1.5±0.05	1.6±0.09	1.4±0.05	1.3±0.05	0.820
**BT roll angle (rad)**	0.6±0.02	0.7±0.02	0.7±0.05	0.8±0.04	0.6±0.02	0.6±0.02	.885
**CB duration (s)**	1.1±0.03	1.0±0.07	1.0±0.03	1.1±0.04	1.0±0.03	1.0±0.03	0.189
**BT duration (s)**	0.8±0.03	0.9±0.08	0.8±0.03	0.8±0.04	0.8±0.03	0.8±0.03	0.643

**Table 4:** Summary of performance data. CB, BT and C_p_ indicate cutback, bottom turn, and power coefficients, respectively. Yaw, roll and pitch rotation rates are calculated from their angles divided by duration. Mean wave power, rotational powers, power coefficients and total power over inertia are calculated using Eqs 1, 3, 5 and 6, respectively. Trace CB power and CB speed were measured by the TraceUp^TM^ datalogger. Raw data is found in S3 File.

	Participant				Fins		
Performance means	1	2	3	4 (WCT)	Control	RW	p-value
**Trace CB power**	2.6±0.1	4.8±0.5	5.2±0.2	6.0±0.3	4.0±0.2	4.0±0.2	0.625
**CB speed (m/s)**	5.7±0.1	7.4±0.3	6.8±0.1	6.9±0.2	6.4±0.1	6.3±0.1	0.873
**CB yaw rate (rad/s)**	2.2±0.1	2.8±0.2	3.0±0.1	3.2±0.1	2.6±0.1	2.7±0.1	0.143
**CB roll rate (rad/s)**	0.9±0.03	1.3±0.1	1.3±0.1	1.4±0.1	1.1±0.04	1.1±0.05	0.094
**CB pitch rate (rad/s)**	0.6±0.03	0.8±0.1	0.8±0.04	0.9±0.1	0.7±0.03	0.7±0.04	0.005
**CB yaw power (W)**	70±5	164±34	222±19	228±37	140±14	163±17	0.035
**CB roll power (W)**	14±2	43±13	47±5	80±26	28±3	36±5	0.011
**CB pitch power (W)**	7±1	18±6	23±4	31±9	13±2	18±4	0.005
**C**_**p**_ **CB**	0.09±0.01	0.09±0.02	0.13±0.01	0.15±0.02	0.11±0.01	0.12±0.01	0.042
**BT initial speed (m/s)**	6.2±0.1	8.8±0.3	7.7±0.2	7.4±0.2	7.1±0.1	7.1±0.1	0.512
**BT yaw rate (rad/s)**	1.7±0.05	1.7±0.1	1.9±0.1	1.9±0.1	1.8±0.05	1.8±0.06	0.631
**BT roll rate (rad/s)**	0.9±0.03	0.8±0.1	0.9±0.03	1.06±0.05	0.9±0.03	0.9±0.03	0.371
**BT yaw power (W)**	59±4	62±9	99±6	74±6	74±4	83±6	0.023
**BT roll power (W)**	21±4	18±7	33±5	41±9	25±4	28±5	0.23
**C**_**p**_ **BT**	0.06±0.003	0.03±0.004	0.05±0.003	0.05±0.004	0.05±0.003	0.05±0.003	0.145
**Total power (W)**	170±10	302±53	419±29	444±77	278±20	326±27	0.005
**Total C**_**p**_	0.35±0.02	0.30±0.05	0.43±0.03	0.45±0.05	0.37±0.02	0.40±0.03	0.076
**Total Power / Inertia (s**^**-3**^**)**	8.3±0.5	10.6±1.9	13.6±0.9	17.1±3.0	10.5±0.7	11.7±0.9	0.026

Speed and distance data were significantly higher for control fins. Mean maximum speeds are considered high speed wave riding [28], with Participants 2, 3, and 4 (WCT) frequently achieving extremely high speeds [28].

Mean wave power during a surf session was 18.8 percent higher for control fins.

**Fig 5:**
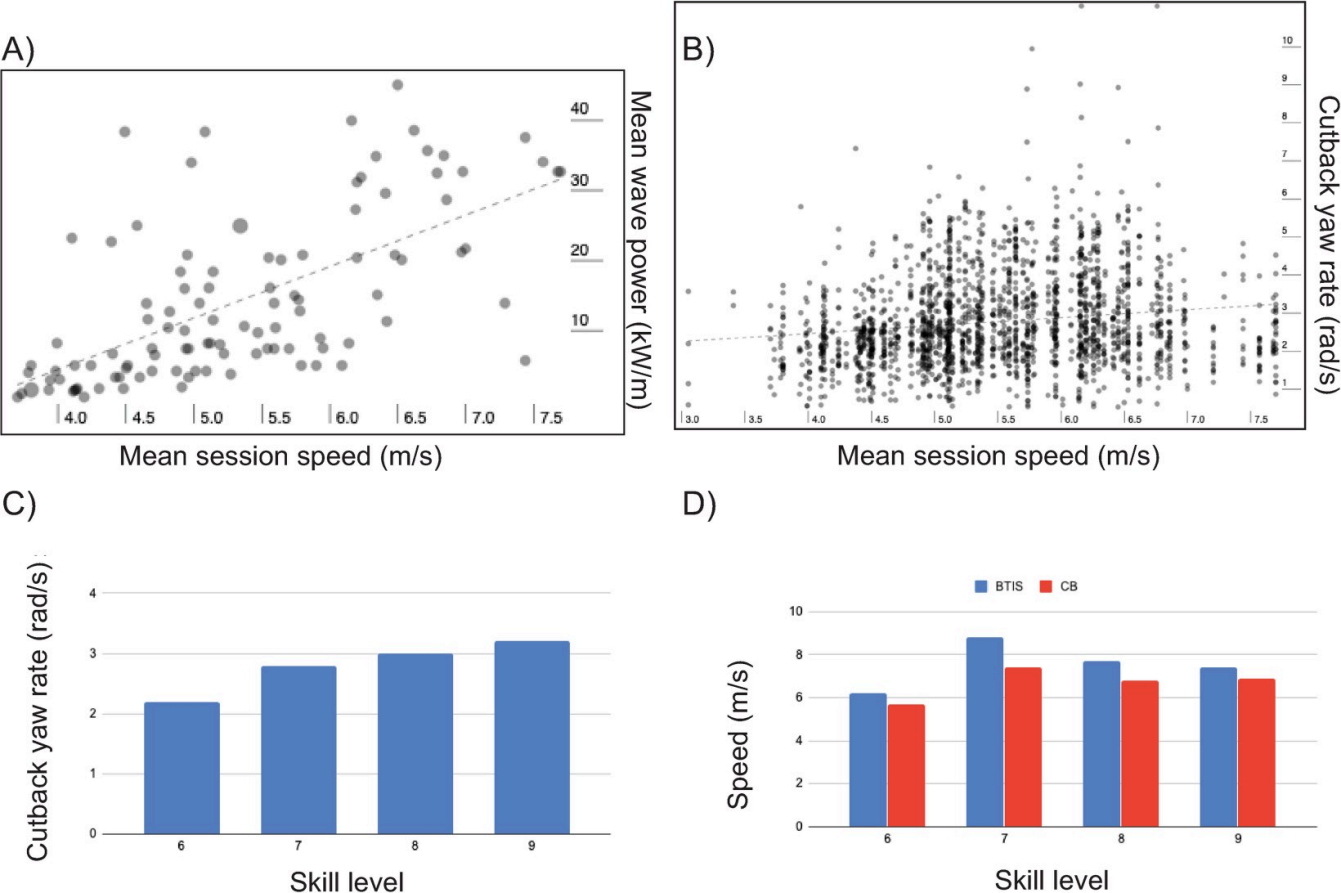
Comparisons of various performance means. (A) Session wave power vs. mean session speed. (B) Cutback yaw rate vs. mean session speed. For both (A) and (B), a positive Pearson correlation exists (p<0.001). (C) Cutback yaw rate vs. Skill level. (D) Bottom turn initial speed(BTIS) and cutback speed vs. Skill level.

Wave height is the most important factor affecting wave power, and we observed a significant, positive Pearson correlation between wave power and surfer speed (Fig 5A), similar to the results of others [27, 43]. Because of the direct correlation, we created a scale factor to facilitate comparison of speed and distance data by using the ratio of mean session speeds (S_s_). The dimensionless scale factor, K_wp_ is therefore

$$K_{wp}=\frac{lowS_{s}}{highS_{s}}$$ (2)

As only two fins were compared, the treatment (RW) and control (C), the scale factor was only applied to the fin with the higher mean WP.

Because of large percent differences in wave powers between control and RW fins, Eq 2 was used to create a scale factor of K_wp_ = 0.949. When applied to the control’s mean max speed, this resulted in a speed of 8.04 m/s, resulting in a 1.2% improvement for RW. Distance still had a large, 10.5% increase for the control fins. The larger percent difference in distance is likely related to the surf break, or location, as some breaks provide longer rides in similar wave energies. For example, for the control, the top two surf breaks were Walter’s West and Changes (Hawaii), with a mean distance of 76.3 m surfed per wave. For RW, the top two surf breaks were Rocky Point and Gas Chambers (Hawaii), with a mean distance of 54.9 m. Rocky Point and Gas Chambers break over 200 m closer to shore, limiting ride distance for obvious reasons.

Also note in Table 3 there is no apparent correlation of distance surfed with skill level, as the skill level 6 surfer is closer to WCT’s mean distance than to either skill level 7 or 8. Another reason for the difference in mean distance could be due to drag, as unpublished CFD results showed RW thruster fins have a 4–9% higher drag than control fins with smooth leading edges.

Mean turn durations in Table 3 were not significantly different between RW and control fins. They also showed no (CB) or low (BT) Pearson correlation with respect to skill level, but significant, positive correlations with respect to wave power. Larger waves can allow more time to carve a turn.

For the five turn angles measured by the TraceUp^TM^ device, only CB pitch was significantly higher for RW compared to the control.

### Fin performance data analysis

To aid in comparing performance of RW vs. control fins, we made the following calculations from the TraceUp^TM^ datalogger measured data found in Table 3 and S3 File. Calculated results are found in Table 4. All data in Table 4 is calculated from the following equations, with the exception of Trace CB power, CB speed, and BT initial speed, which were all measured by the tracking system. Rotation rates (ω) were calculated per [34] by dividing the cutback and bottom turn angles by their respective durations. Some obvious outliers were observed, where rotation rates were unreasonably high for a surfer to accomplish. Outliers were defined as values greater than two standard deviations from the mean for Participant 4, the WCT surfer. Using the WCT surfer as a baseline also allowed us to define a reasonable minimum cutback yaw rate of 0.53 radians per second. Using these upper and lower limits eliminated under 4% of the data.

Rotational power (P) was calculated using:

$$P=\frac{0.5I\omega^{2}}{t}$$ (3)

where *I* = moment of inertia in kg•s found in Table 1 and calculated per [34], *ω =* pitch/yaw/roll rate of the cutback or bottom turn in rad/s, and *t* = turn duration in s. A dimensionless power coefficient, *C*_*p*_, was then calculated by dividing by a speed-related power value. While wave power predictions using Eq 1 were recorded for participants 1–3, they were not recorded for Participant 4. However, because of the positive correlation between wave power and surfer speed, we used mean session speed to calculate what we refer to as a session speed wave power, *P*_*w*_. A “session” is the duration of an individual surfing trip recorded by Trace, so the mean session speed correlates positively with predicted wave power during that time frame (Fig 5A). Session speed wave power could therefore serve as a predictor of actual wave power during the surf session.

The session speed wave power was calculated using:

$$P_{w}=\frac{0.5m(S_{s}{)}^{2}}{t}$$ (4)

where *m* = mass of surfer+board, *S*_*s*_ is mean session speed, and *t* is the duration of cutback or bottom turn. Dividing Eq 3 by Eq 4 cancels both 0.5 and t, yielding

$$C_{p}=\frac{\Sigma (I\omega^{2})}{m(S_{s}{)}^{2}}.$$ (5)

For cutbacks, *Σ(Iω*^*2*^*)* included the sum of cutback roll+pitch+yaw powers, while bottom turn coefficients included the sum of roll+yaw.

As Fig 5B shows, a small, positive correlation exists between mean session speed and cutback rotation rate. Eq 5 was therefore a way for us to compare RW and C, regardless of wave power and surfer+board mass. Similar reasoning is used with other dimensionless coefficients. For example, lift coefficients allow for the comparison of different wings regardless of dynamic pressure and wing type [48].

It is also interesting to attempt to eliminate the effects of inertia, as inertia takes into account the dimension of the surfboard and the combined mass of surfer and surfboards [34]. In other words, surfers of similar weight will have more inertia on a longer board compared to a shorter board. In similar fashion, a heavier surfer on a longer and wider board will have more inertia compared to a lighter surfer on a shorter and narrower board. While not dimensionless, P/I, with units of s^-3^, was calculated

$$P/I=\frac{0.5\omega^{2}}{t}$$ (6)

as a measure of total cutback or bottom turn power, divided by moment of inertia found in Table 1. Note that C_p_ also partially eliminates inertia effects by canceling mass of surfer+board.

Table 4 lists performance data for 18 means used for analyzing surfer performance during the cutback and bottom turn. Comparing participant skill level, ANOVA revealed p < 0.001 for all performance means. Therefore, significant differences due to skill level existed for all performance means. All but two performance means showed significant, positive Pearson correlations with respect to skill level, with *C*_*p*_ *yaw BT* having a negative correlation, and *total C*_*p*_ having no correlation. No correlation of *total C*_*p*_ with skill level suggests the coefficient may be useful for future research on fin performance using surfers of different skills and surfing in different ocean conditions. Also, the negative correlation of *C*_*p*_ *yaw BT* with skill level suggests a lower value is more favorable for WCT-level surfing. Perhaps, extracting too much power during the bottom turn results in less power available for the cutback.

A comparison of various summary data points versus skill level and mean session speed is shown in Fig 5. The data shows cutback yaw rate varied directly with skill level (Fig 5C). In Fig 5D, note bottom turn initial speed was always higher than cutback speed, regardless of skill level, also evident in Fig 2.

Comparing the control and RW fins, 16 of 18 performance means (89%) were higher for RW, 44% being significantly higher. Only *CB speed*, and *BT initial speed* were 1.5% and 1.0% lower for RW, respectively. Participant data showed a positive correlation between wave power and cutback speed, and when the scale factor of K_wp_ = 0.949 was applied, mean RW cutback speed was 4.7% greater. Applying K_wp_ to BT initial speed yielded a 4.1% increase for RW. Some of the highest angles of attack the fins will experience occur during a turn, especially a cutback. Compared to designs with smooth leading edges, RW designs have their biggest gains in lift and efficiency at high angles of attack [6].

Rotation rates were all higher for RW, although not always significantly higher. While the improvements may be related to the improved efficiency suggested in [6], it could also relate to RW’s lower mean wave power, as slightly smaller waves may be easier to perform a rapid, but less powerful maneuver on. However *CB speed* (Table 4) and CB yaw angle (Table 3) were nearly identical for both fins, suggesting that the effect of wave size was not important during the cutback.

Except for CB pitch rate, all of the statistically significant differences were related to power or C_p_. The coefficients help to non-dimensionalize the data with respect to wave power and surfer+board mass, while the power values give a clearer picture of what individual surfers are capable of doing with their combination of wave+board+fin. Some of RW’s largest improvements occur during the cutback, which in surfing contests is the maneuver judges award points for. A powerful cutback is typically going to receive more points than a weaker one. The cutback yaw power shows the largest increase for RW, 23 W, a 16.4% improvement. Cutback roll and pitch power have smaller total power gains, but bigger percent gains.

Surfing requires balancing skills, with any slight loss of control resulting in wasted energy. Board control is critical. Flow imagery from cameras attached to surfboards revealed typical conditions involve rapid, ±10° changes in flow direction relative to the fins [27]. Perhaps, improvements in RW’s power values are related to improved flow control, resulting in a more stable ride that allows for more effective power transfer. In separate reviews of tubercle applications [3, 39], tubercled leading edges were found to reduce unsteady fluctuations and tonal noise, thereby improving efficiency. In a numerical study using an upstream cylinder to generate turbulence, Tong *et al*. [49] discovered tubercled leading edges provided a substantial reduction in lift and drag fluctuations. Considering the humpback whale, it makes sense their fins are designed to extract as much power as possible during a maneuver, optimizing stability amidst turbulent flow. Also, for stealth purposes, minimizing pressure pulses is an obvious advantage while feeding. And, like a surfer, a humpback’s maneuvers are extremely dynamic, with big changes in *θ* on all three axes, but also d*θ/*d*t* and d^2^*θ/*d*t*^2^.

The present study is the first to present evidence of dynamic roll, pitch and yaw performance of tubercled designs. Research on dynamic behavior of tubercled designs is currently limited only to sustained, sinusoidal flapping (roll+pitch) or pitching motions more frequently associated with humpback flukes, not flippers [21]. Observations of wild humpbacks by the present authors indicate that flipper pitching motions occur most frequently in combination with yaw motions, as they rapidly rotate their fins forward to generate lift. Brief pectoral flapping motions are used when a humpback wants to accelerate quickly, usually vertically, as during lunge feeding [50].

Flapping motion studies show mixed results. Stanway’s study [12], using the same modified flipper designs as [13–15], found tubercled leading edges degraded flapping performance. However, the experiment was carried out at Re near 1•10^5^, a value Johari [15] found to have poor static performance for the same flipper models. A pitching study by Wang *et al* [51] found a negligible change in performance in tubercled designs, although foil geometry was quite different than [12], and Re was an order of magnitude greater. In contrast, a study by Zhang et al [52] revealed potential advantages for tubercled leading edges during flapping flight.

Both [12] and [51] studied sustained pitching motion over ranges as much as ±20° *α*, something humpback whales, or surfers, would likely never do. Both are more likely to perform short duration, high d^2^θ/dt^2^ pitch changes, where interference with the reverse Karman vortex street, as Stanway [12] suggested, would not occur or be minimal.

Surfboard pitch corresponds to surfing fin yaw, and our study found a 45.2% increase in cutback pitch power for RW. While no current studies provide data on dynamic roll/pitch/yaw changes, many studies have been performed on yaw, or rotation rates. As wind and water turbines, many studies show improvements in efficiency and power coefficients for tubercled leading edges [37, 53–54], as well as RW designs [4]. Improvements are also noticed for tubercle applications to propellers [55–56]. For RW designs tested in [4], cut-in velocity decreased, possibly because of RW’s reduced moment of inertia. Hansen [38] was the first to identify reductions in tonal noise from tubercle applications. Summaries [3] and [39] also found many examples for both fixed and rotating shapes.

In our study, RW’s cutback roll power increased by 28.6%. Currently, no research exists on dynamic roll behavior of tubercled designs. However a static roll experiment by Wei et al [57] found their tubercled design yielded large performance gains and delayed stall in roll. Their experiment studied roll over a 60° range, with the wing fixed at 20° *α* and 15° sweep. Flow visualization showed that, at high roll angles (+15°), the tubercles limit the wingtip vortex and associated stall effects.

### Fin performance relative to a top-ranked professional

The data in Table 4 suggests that some of our participants were able to generate more power when using RW designs. This could result in improved surfing experiences for recreational surfers, but also increased performance for competitive surfers.

For example, for Participant 2 (Skill Level 7 surfer), 41% of the performance data shows no significant difference compared to that of Participant 4 (the WCT surfer). The most interesting results are for the Skill Level 8 surfer (Participant 3), whose percentage doubled from 41 to 82% as a result of using RW fins. Of those results, 41.2%, were higher than the Participant 4 (WCT) surfer means, with 2 values related to bottom turns being significantly higher, *C*_*p*_ *yaw BT* and *BT yaw power*. However, as noted earlier, a higher *C*_*p*_ *yaw BT* may not be preferable. Also, *C*_*p*_ *yaw BT* may be influenced by other factors, including surf break and wave height and power. As expected, all of the Skill Level 6 means were significantly lower than the WCT surfer, regardless of fin type.

At the time of data collection, participant 4 (WCT surfer) was ranked at the top of the WSL Men’s Championship Tour. Although more research is needed, these results show that by switching to RW fins, it may be possible for a highly skilled surfer to gain a competitive advantage. Dynamic CFD research, similar to that of Oggiano and Pierella [58] that incorporates the air/water interface, may be useful in gaining a deeper understanding of performance differences observed in the field. We also envisage that testing our designs under ideal wave conditions (e.g. a wave pool that generates identical waves) would eliminate the influence of variability in wave power at ocean-based surf locations. In a wave pool, Eq 6 may be more useful for comparing fin performance.

## Conclusions

We conducted field analysis on ocean waves for the purpose of studying a novel, top-down approach to applying humpback flipper passive flow control to fins for surfboards. Several issues related to tubercles have been noted in the literature [3, 39], such as, decreased performance under pre-stall angles of attack and the correct selection of tubercle amplitude and wavelength. While these issues are certainly important, perhaps the bigger issue is the failure to use a more top-down approach to humpback flipper applications. Efforts to more closely mimic the actual humpback flipper, with its many passive flow control features (besides tubercles), results in complex patterns that are difficult to manufacture and costly to prototype using conventional methods. However, rapid prototyping through Additive Manufacturing (e.g. 3D printing) can overcome these manufacturing challenges and the results presented in this paper suggests the added complexity may be worth it for the resulting increased surfing performance.

Shortboards fitted with RW designs showed a wide-spread increase in performance, i.e. 89% of the performance means analyzed resulted in an improvement, with 44% of these being statistically significant. In particular, statistical analysis revealed significant differences between skill levels, except for *total C*_*p*_, suggesting this coefficient might be useful in future work involving different skill levels surfing in variable ocean conditions.

It was demonstrated that using RW designs can improve a surfer’s performance. For example, the performance of a Skill Level 8 surfer using control fins is similar compared to a Skill Level 9 (WCT) surfer in the minority (41%) of the analyzed performance means. In contrast, the similarity increases to 82% when the Skill Level 8 surfer uses RW fins. This indicates that it may be possible for surfers to obtain a performance advantage in competitive surfing using RW fin designs.

## Supporting information

S1 Video Drone footage captured by David Shormann with overlay of performance data using the Trace Video App. (MP4)

S2 Video Drone footage captured by David Shormann with overlay of performance data using the Trace Video App. (MP4)

S3 Video Drone footage captured by David Shormann with overlay of performance data using the Trace Video App. (MP4)

S1 File Raw session data for Table 3 means. Includes TraceUp^TM^ measured mean session speed, wave power calculated per Eq 1, and surf session location.
(CSV)

S2 File Raw wave data for Table 3 means. Includes TraceUp^TM^ measured max speed and distance surfed on an individual wave.
(CSV)

S3 File Raw Cutback (CB) and Bottom Turn (BT) data for Tables 3 and 4 means. Includes TraceUpTM measured turn angles and durations, with means found in Table 3, measured turn speeds and Trace CB power, with means found in Table 4, and calculated rotation rates, powers and power coefficients, calculated per Eqs 4–6, with means found in Table 4.
(CSV)
